# Time‐Efficient Interleaved Spin Density‐Weighted and Inversion Recovery ^23^Na MRI of the Human Calf Muscle at 7 T

**DOI:** 10.1002/mrm.70536

**Published:** 2026-07-21

**Authors:** Tobias Wilferth, Fiona Meyer, Jordan M. Höhn, Michael Uder, Armin M. Nagel

**Affiliations:** ^1^ Institute of Radiology, University Hospital Erlangen Friedrich‐Alexander‐Universität Erlangen‐Nürnberg (FAU) Erlangen Germany; ^2^ Division of Medical Physics in Radiology German Cancer Research Centre (DKFZ) Heidelberg Germany

**Keywords:** ^23^Na MRI, 7 T, interleaved MRI, inversion recovery MRI, ultra‐high field strength

## Abstract

**Purpose:**

Spin density‐weighted (SDW) and inversion recovery (IR) ^23^Na MRI provide different sodium contrasts with complementary information. Therefore, the aim was to develop a time‐efficient sequence scheme capable of providing both contrasts by acquiring SDW and IR ^23^Na MRI data within a single sequence without additional measurement time.

**Methods:**

In the developed pulse sequence, the conventional 180° inversion pulse was divided into two successive 90° pulses, with an additional readout inserted between them. The sequence was initially evaluated in simulations and phantom measurements. Afterwards, it was applied in calf muscle measurements of six healthy volunteers to assess its practical feasibility and signal characteristics. All ^23^Na measurements were acquired at 7 T and compared to standard SDW and IR sequences.

**Results:**

The interleaved SDW/IR sequence yielded image quality and contrast comparable to standard SDW and IR sequences. Phantom experiments showed slightly higher remaining *T*_1_‐weighting in the interleaved sequence compared with standard SDW, resulting in only minor changes in image contrast. Fluid suppression was effective across all IR approaches, though efficiency decreased modestly with longer effective inversion pulse lengths. Spin dynamic simulations for IR ^23^Na MRI indicated slightly increased sensitivity to fluid suppression artifacts arising from *B*_0_ inhomogeneities in the interleaved sequence. In vivo calf muscle measurements did not reveal significant differences in the mean muscle signal between the interleaved SDW/IR sequence and the corresponding standard sequences.

**Conclusion:**

The proposed interleaved sequence enables simultaneous acquisition of ^23^Na SDW and IR contrasts, thereby reducing overall scan time and eliminating the need for co‐registration.

## 1 Introduction

Sodium (^23^Na) MRI is established as a noninvasive technique to get insights into tissue ion homeostasis and numerous studies have demonstrated the potential for clinical research in several diseases [1, 2, 3, 4]. Spin density‐weighted (SDW) ^23^Na MRI enables determination of the apparent tissue sodium concentration (aTSC), which represents a volume‐weighted average of intra‐ and extracellular sodium [5]. Inversion recovery (IR) ^23^Na MRI further exploits differences in longitudinal relaxation to suppress signals from specific compartments [6, 7, 8]. Since pathologies can alter total sodium content, distribution, and relaxation times, SDW and IR acquisitions provide complementary insights into sodium homeostasis and cellular metabolism [4]. In particular, IR ^23^Na measurements of patients with muscular channelopathies suggest that IR sequences allow for a weighting toward the intracellular sodium signal [8]. Moreover, the joint application of SDW and IR ^23^Na MRI measurements for human calf muscle was shown to enable additional insights into muscular dystrophies, for example Duchenne [9, 10] and Facioscapulohumeral muscular dystrophy (FSHD) [11].

The major challenges in ^23^Na MRI are low spatial resolution and long acquisition times due to reduced signal‐to‐noise ratio (SNR) and lower in vivo ^23^Na concentrations compared to ^1^H MRI. To reach a reasonable SNR with nominal isotropic spatial resolutions of about 3 mm, common acquisition times for a single ^23^Na MRI contrast are typically in the range of 10–15 min. ^23^Na IR MRI provides even lower SNR compared to ^23^Na SDW MRI due to the magnetization preparation required to suppress the MR signal of specific compartments. Consequently, although combining standard SDW and IR ^23^Na MRI measurements in one protocol would be desirable due to the complementary information, it is often hindered in clinical research applications due to lengthy scan times resulting from the low SNR of ^23^Na MRI.

To increase SNR in ^23^Na IR MRI, prolonged inversion pulse durations have been employed for fluid suppression in brain, muscle and cartilage imaging [7, 12, 13]. Furthermore, these approaches have been shown to reduce specific absorption rate (SAR) limitations and to provide improved contrast for human calf muscle ^23^Na IR MRI. However, this comes at the cost of increased sensitivity to *B*_0_ inhomogeneities resulting in suppression artifacts.

To increase the scan time efficiency of ^23^Na MR measurements, interleaved acquisitions where the idle time resulting from ^23^Na magnetization recovery or preparation is used for the acquisition of different contrasts are promising. For example, ^23^Na MRI has been interleaved with ^1^H MRI to obtain data for motion correction [14, 15], tissue segmentation [15] or further anatomical examinations [16] and tissue parameters [17]. Furthermore, different ^23^Na contrasts can be acquired interleaved, as shown for SDW and triple quantum filtered ^23^Na MRI [18]. Although this approach does not reduce the scan time for the single contrast, the total scan time for the whole MR protocol can be shortened considerably.

The purpose of this work was to combine SDW and IR ^23^Na MRI in a time‐efficient interleaved manner within a single pulse sequence by splitting a long 180° inversion pulse into two shorter 90° pulses and inserting the SDW readout in between. The sequence then provides both contrasts simultaneously within the scan time of the ^23^Na SDW sequence addressing the problem of prolonged scan times originating from the low SNR inherent to ^23^Na MRI. We demonstrate the feasibility of interleaved SDW and IR ^23^Na MRI in phantom and in vivo measurements of human calf muscle and compare the developed SDW/IR ^23^Na MRI pulse sequence to standard SDW and IR ^23^Na MRI pulse sequences.

## 2 Methods

All ^23^Na MR measurements were performed on a whole‐body 7 T MR system (MAGNETOM Terra.X, Siemens Healthcare, Erlangen, Germany) using a dual‐tuned ^23^Na/^39^K leg radiofrequency (RF) coil (RAPID Biomedical, Rimpar, Germany). At the beginning of every measurement, a global flip angle calibration and manual *B*_0_ shim based on ^23^Na *B*_0_ maps were applied [19]. All ^23^Na MR images were acquired using a density‐adapted 3D radial readout scheme [20]. Afterwards, a custom‐written MATLAB R2022b tool (MATLAB, The MathWorks, Natick, MA, USA) was used to reconstruct the acquired radial raw data sets offline. A Hamming filter was used to increase SNR and to reduce Gibbs' ringing artifacts [21, 22]. A density compensation was applied before performing a nonuniform Fast Fourier Transform (NUFFT) [23] with a two‐fold grid oversampling.

All examined subjects provided informed consent prior to the MRI examination and the in vivo MRI measurements were approved by the local ethical review board.

### 2.1 Interleaved SDW and IR Sequence

The standard SDW and IR sequences, as well as the newly developed interleaved SDW/IR sequence, are schematically illustrated in Figure 1A along with all relevant parameters. In the SDW/IR sequence, the 180° inversion pulse is divided into two 90° pulses, with an intermediate readout inserted between them to generate the SDW contrast. After each readout, a rewinder gradient is applied to refocus the transverse magnetization. This is especially important after the SDW readout to complete the inversion by the following RF pulse. The second 90° pulse completes the inversion, and after the inversion time (TI), a third 90° pulse is followed by a readout acquiring the IR contrast analogous to the standard IR sequence. Finally, the remaining transverse magnetization is spoiled using a trapezoidal spoiler gradient in the *z*‐direction with a spoiling factor of 4*π*. Furthermore, RF spoiling with a 50° phase increment per TR is applied to both RF pulse and readout phases. The sequence allows for the acquisition of the SDW and the IR signal using different nominal spatial resolutions. For the standard IR sequence, TI was defined as the time between the middle of the inversion pulse and the middle of the excitation pulse. For the new interleaved SDW/IR sequence, the center of the inversion pulse was defined as the temporal midpoint between the two 90° pulses.

**FIGURE 1:**
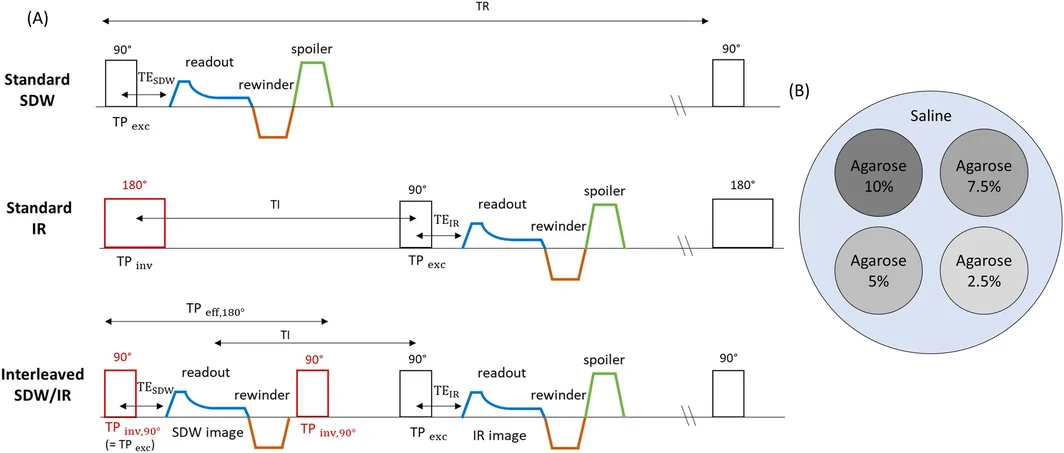
(A) Schematic of the standard SDW and IR sequences as well as the new interleaved SDW/IR sequence. In the interleaved sequence, a second 90° pulse after the SDW readout completes the first 90° pulse to the effective 180° inversion. For all readouts, density‐adapted 3D radial readout gradients are applied followed by trapezoidal rewinder gradients with equal gradient moment and opposite polarity to refocus the magnetization. At the end of every TR, the remaining transverse magnetization is spoiled using a trapezoidal spoiler gradient. (B) Phantom with five compartments containing 100 mM NaCl and different agarose concentrations (10%, 7.5%, 5%, 2.5%, and 0%).

### 2.2 Spin Dynamic Simulations

The spin dynamic simulations of the spin‐3/2 ^23^Na nuclei were performed with a custom‐written MATLAB R2022b software tool [7, 13, 24] and used to investigate the evolution of the longitudinal magnetization during the inversion and to confirm the IR measurement results with respect to the influence of *B*_0_ inhomogeneities. The simulation is based on the differential equations describing the evolution of the density operator expressed by irreducible spherical tensor operators [25, 26]. The simulated MR pulse sequence is separated into arbitrary time intervals and for every time step the matrix equation determining the spin dynamics of the systems is solved by matrix diagonalization [25]. In addition to the sequence characteristics, the spectral densities *J*_0_, *J*_1_, and *J*_2_ and the off‐resonance *ω*_*off*_ served as input parameters. The spectral densities were determined in separate phantom measurements for saline solution (*J*_0_ = *J*_1_ = *J*_2_ = 8.8 Hz), agarose 5% (*J*_0_ = 163 Hz, *J*_1_ = 15.5 Hz, *J*_2_ = 12.9 Hz) and agarose 10% (*J*_0_ = 321 Hz, *J*_1_ = 25.7 Hz, *J*_2_ = 17.3 Hz) using spatially non‐selective measurements of the signal evolution of spin echo and IR sequences of spherical phantoms containing only the single compartment [13].

### 2.3 Phantom Measurements

A five‐compartment phantom (see Figure 1B) was used to evaluate the interleaved SDW/IR sequence and compare the results to standard SDW and IR sequences, in particular with regard to the influence of *B*_0_ inhomogeneities as well as the alteration of image contrast between compartments with different relaxation characteristics. Agarose gels of different concentrations (2.5%, 5%, 7.5%, and 10%) were filled in cylindrical sectors and the space in between was filled with saline solution. All compartments had a ^23^Na concentration of 100 mM. The saline solution compartment represented the fluid compartment and was aimed to be suppressed in the IR measurements.

To evaluate the interleaved SDW/IR sequence, measurement results were compared to the standard SDW and IR sequences. The following sequence parameters were kept the same for all ^23^Na MRI phantom measurements: nominal spatial resolution (Δ*x* ^3^)_SDW_ = (Δ*x* ^3^)_IR_ = (4 mm)^3^, echo time TE_SDW_/TE_IR_ = 0.3 ms, 90° excitation pulse length TP_exc_ = TP_inv,90°_ = 0.5 ms and readout time T_RO_ = 5 ms.

Standard IR measurements were acquired for inversion pulse lengths TP_inv_ of 1 and 6.5 ms. The 6.5 ms is the shortest possible effective inversion pulse length TP_eff,180°_ of the interleaved SDW/IR sequence covering two 90° pulses with TP_inv,90°_ = 0.5 ms and the 5 ms SDW readout including readout and rewinder gradients. The measurements were performed with repetition times TR = 150 ms (acquisition time *T*_AQ_ = 10 min, TI = 34 ms) and TR = 360 ms (*T*_AQ_ = 24 min, TI = 38 ms), where the latter was used to compare the interleaved and the standard SDW data sets without steady‐state effects from incomplete *T*_1_‐relaxation.

### 2.4 In Vivo ^23^Na Calf Muscle Measurements

In vivo measurements of the left human calf were performed in six healthy volunteers (three female, three male, age 27 ± 4 years). For all volunteers, the results of the interleaved SDW/IR sequence (TR = 150 ms, TP_exc_ = TP_inv,90°_ = 0.5 ms, TI = 34 ms, TE_SDW_/TE_IR_ = 0.3 ms, *T*_RO_ = 5 ms, nominal spatial resolutions (Δ*x* ^3^)_SDW_ = 4 × 4 × 4 mm^3^ and (Δ*x* ^3^)_IR_ = 5 × 5 × 5 mm^3^, *T*_AQ_ = 12:30 min) were compared to the results of the standard SDW sequence (TR = 150 ms, TP_exc_ = 0.5 ms, TE_SDW_ = 0.3 ms, *T*_RO_ = 5 ms, nominal spatial resolution (Δ*x* ^3^)_SDW_ = 4 × 4 × 4 mm^3^, *T*_AQ_ = 12:30 min) and the standard IR sequence (TI = 34 ms, TR = 150 ms, TP_exc_ = 0.3 ms, TE_IR_ = 0.3 ms, *T*_RO_ = 5 ms, nominal spatial resolution (Δ*x* ^3^)_IR_ = 5 × 5 × 5 mm^3^, *T*_AQ_ = 12:30 min). The standard IR sequence again was measured with inversion pulse lengths TP_inv_ of 1 and 6.5 ms.

In the quantitative analysis, in addition to the whole muscle, two different muscle ROIs were used since earlier studies suggested that relative signal intensities of multiple quantum filtration sequences vary between different muscle groups [27]. As it was not possible to acquire anatomical ^1^H MRI data for every volunteer, the ROIs were drawn on the standard SDW ^23^Na image and used for all acquired images. For this purpose, the ^23^Na IR k‐space data were zero‐filled to the 4 mm isotropic voxel size of the ^23^Na SDW data. Both ROIs were chosen in a way that the main arteries and bones were excluded. ROI 1 corresponds to the gastrocnemius lateralis, the gastrocnemius medialis, and the soleus muscle. ROI 2 corresponds to the tibialis anterior, the peroneus longus, and the extensor digitorum longus muscle. The signal intensities of the SDW images were corrected for the higher spatial resolution by the factor (Δ*x* ^3^)_IR_/(Δ*x* ^3^)_SDW_.

All statistical analyses were done with MATLAB R2022b, and results were considered statistically significant for *p* values < 0.05. The differences between the mean signal intensities obtained by the different sequences were tested for significance using the Friedman test with post hoc Bonferroni correction for multiple comparisons. Only statistically significant *p* values are presented in the text, and the given values have already been corrected for multiple comparisons.

## 3 Results

### 3.1 Phantom Measurements and Spin Dynamic Simulations

The phantom measurement results for the standard SDW and IR sequences as well as the interleaved SDW/IR sequence with a TR of 150 ms are shown in Figure 2. Compared to the standard SDW image, the SDW image of the interleaved SDW/IR sequence presents slightly reduced relative signal values depending on the relaxation behavior of the compartment (agarose 10% with 96% to saline with 91%) causing a minor change of image contrast. The standard IR pulse with TP_inv_ = 1 ms demonstrates very good fluid suppression with a relative signal of only 8.4% compared to the standard SDW sequence. For the increased inversion pulse length TP_inv_ = 6.5 ms, the relative saline solution signal rises to 11.9%. The corresponding IR image of the interleaved sequence shows a relative saline signal of 13.4%. Furthermore, the IR signals of the non‐suppressed compartments increase with increasing IR pulse duration and are slightly higher for the interleaved IR sequence compared to the standard IR sequence under matched effective inversion pulse lengths.

**FIGURE 2:**
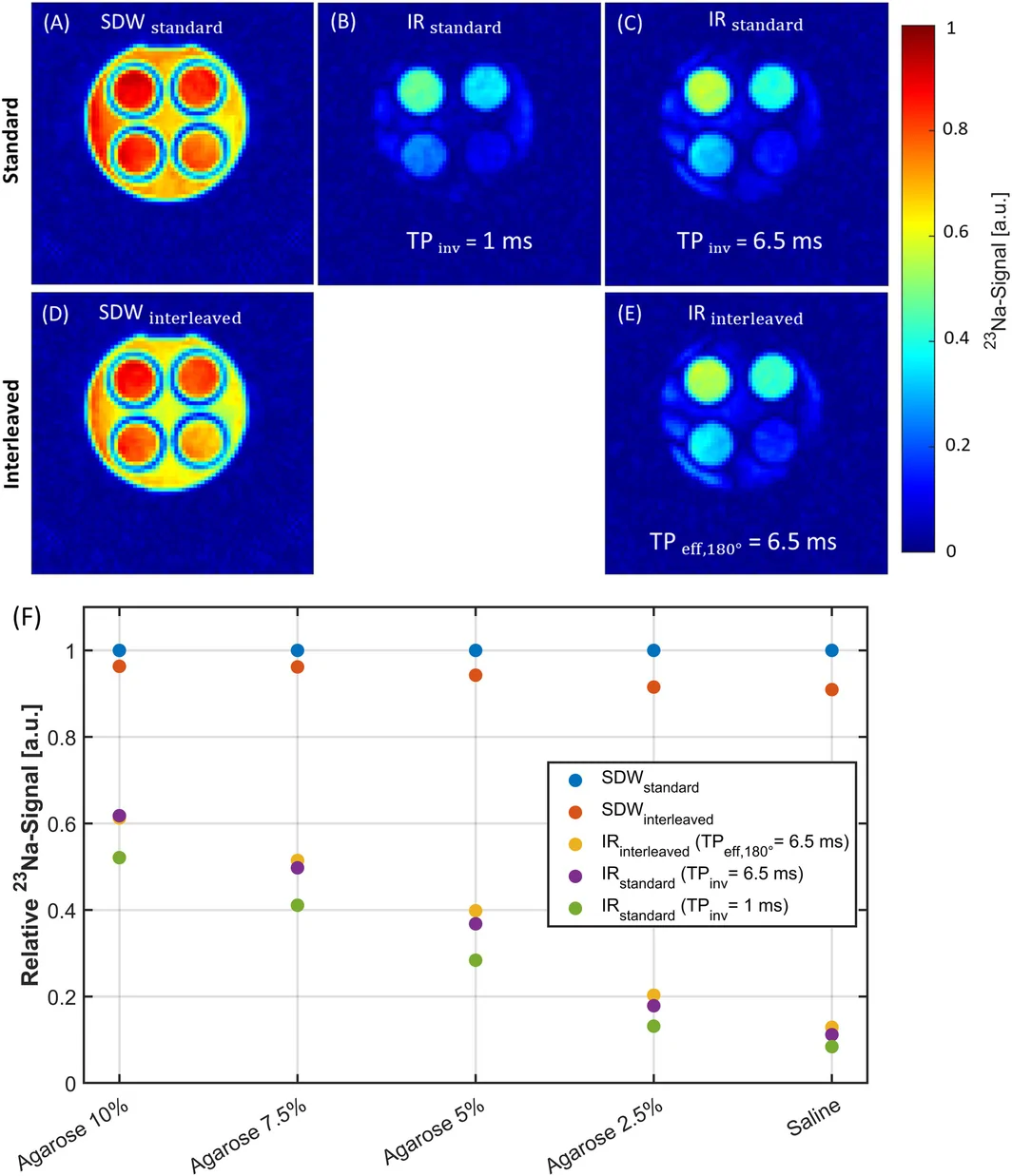
Phantom measurement results for all investigated sequences with TR = 150 ms. Compared to the standard SDW image (A), the interleaved SDW image (D) presents minor compartment‐dependent signal reduction causing a slightly changed image contrast. All IR images (B, C, E) show effective fluid suppression. Longer pulses result in minor suppression artifacts and increased signal for non‐suppressed agarose compartments. Both effects are slightly more prominent in the interleaved IR image (E) compared to the standard IR image (C). (F) The relative ^23^Na signal normalized to the values of the standard SDW image is shown for all phantom compartments for all investigated sequences (TR = 150 ms, TP_inv_ = 1 ms/6.5 ms, TP_eff,180°_ = 6.5 ms). Compared to the standard SDW image, compartment‐dependent signal reduction in the interleaved SDW image leads to a slightly changed image contrast. The IR signals increase with longer IR pulse duration and are slightly higher for the interleaved IR sequence compared to the standard IR sequence.

For TR = 360 ms (see Figure S1), only negligible signal differences are observed between the standard SDW and the interleaved SDW images and the relative signals of the IR images are almost identical. Furthermore, only negligible effects of the SDW readout and the corresponding rewinder gradients on the IR image were observed for the interleaved SDW/IR sequence. These effects were in the range of the deviations between two identical IR scans of the interleaved SDW/IR sequence (see Figure S2).

In agreement with the phantom measurement data, simulation results suggest increased relative signal for non‐suppressed compartments in interleaved IR images compared with standard IR images with matched effective inversion pulse lengths, arising from different relaxation characteristics (see Figure 3). While the longitudinal magnetization of saline at the end of the inversion remains nearly identical for the standard and interleaved sequence (−88.9%/−88.8% for standard/interleaved sequence), faster relaxing agarose compartments show larger differences (agarose 5%: −60.1%/−51.7% for standard/interleaved sequence; agarose 10%: −37.0%/−33.6% for standard/interleaved sequence) due to both *T*_1_ recovery and *T*_2_* relaxation between the two 90° RF pulses. In particular, compartments with faster *T*_1_ recovery and *T*_2_* relaxation exhibit a slight increase in transverse magnetization during the second 90° RF pulse, followed by a decrease as the longitudinal magnetization is inverted (i.e., *M*_*z*_ < 0). Therefore, the difference in longitudinal magnetization between the interleaved and standard IR with matched effective inversion pulse lengths at the end of the inversion process depends on the *T*_1_ as well as *T*_2_* relaxation characteristics of the compartment. Consequently, as the difference also translates into a higher longitudinal magnetization of the non‐suppressed compartments after the inversion time TI, the interleaved sequence yields slightly higher IR signal for these compartments.

**FIGURE 3:**
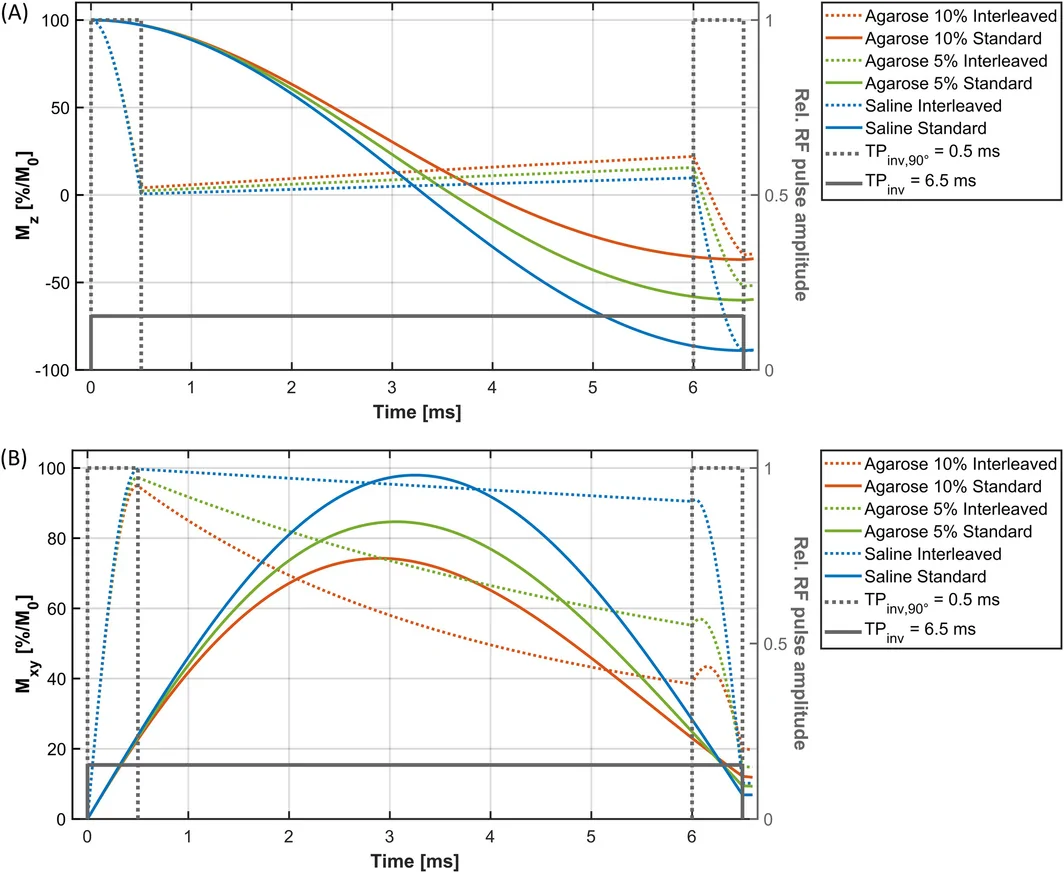
Simulated evolution of the ^23^Na longitudinal magnetization (A) and the transverse magnetization (B) during the standard IR pulse with TP_inv_ = 6.5 ms (solid line) as well as the split inversion pulse consisting of two 90° pulses and separated by the SDW readout also resulting in an effective inversion pulse length TP_eff,180°_ = 6.5 ms (dashed line). In addition to the evolution of the ^23^Na magnetization, the corresponding RF pulse amplitudes are shown in both plots.

Furthermore, also in agreement with the phantom measurement data, simulation results show increased influence of *B*_0_ inhomogeneities on the fluid suppression for the interleaved SDW/IR sequence (see Figure 4).

**FIGURE 4:**
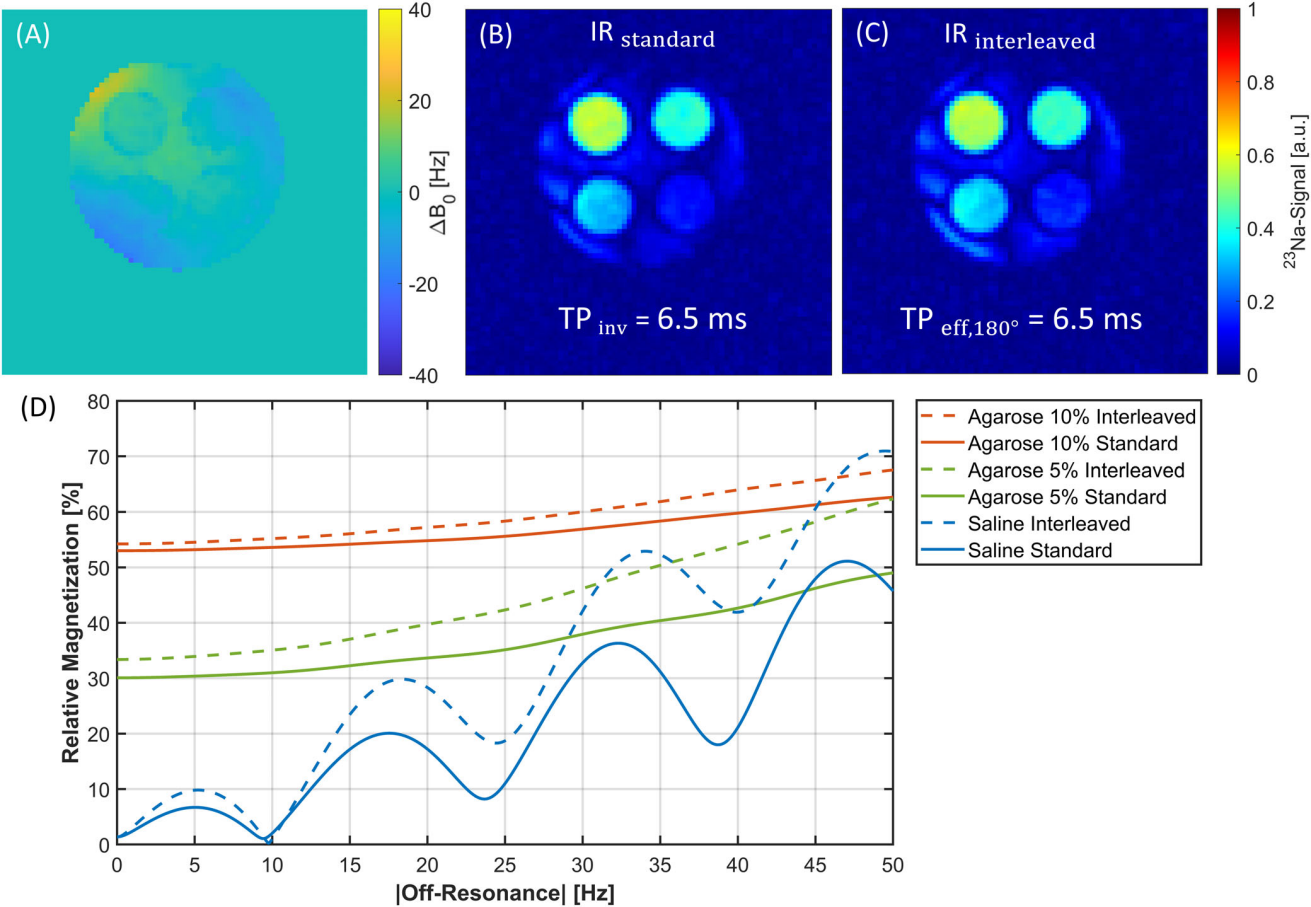
The off‐resonance map (A), calculated from two echoes of the standard SDW sequence, shows off‐resonances between −30 and 30 Hz. Suppression artifacts in the standard IR (B), and slightly more pronounced in the interleaved IR (C), correspond well with the off‐resonance distribution and the simulated relative signal values (D). The simulation, performed for an effective inversion pulse length TP_eff,180°_ = 6.5 ms, also indicates slightly increased signals for the interleaved sequence in agarose compartments (see Figures 2 and 3).

### 3.2 In Vivo ^23^Na Calf Muscle Measurements

Representative measurement results of volunteer Female 03 are shown in Figure 5 for the interleaved SDW/IR sequence as well as the standard SDW and standard IR (TP_inv_ = 1 ms/6.5 ms) sequences. Signal originating from the reference phantoms with saline solution is well suppressed in all IR measurements with only minor suppression artifacts that are slightly more pronounced at longer effective inversion pulse lengths. The IR measurement results of all volunteers can be found in Figure S3. Due to scan time restrictions, for volunteer Male 02 no standard IR measurement with TP_inv_ = 6.5 ms is available.

**FIGURE 5:**
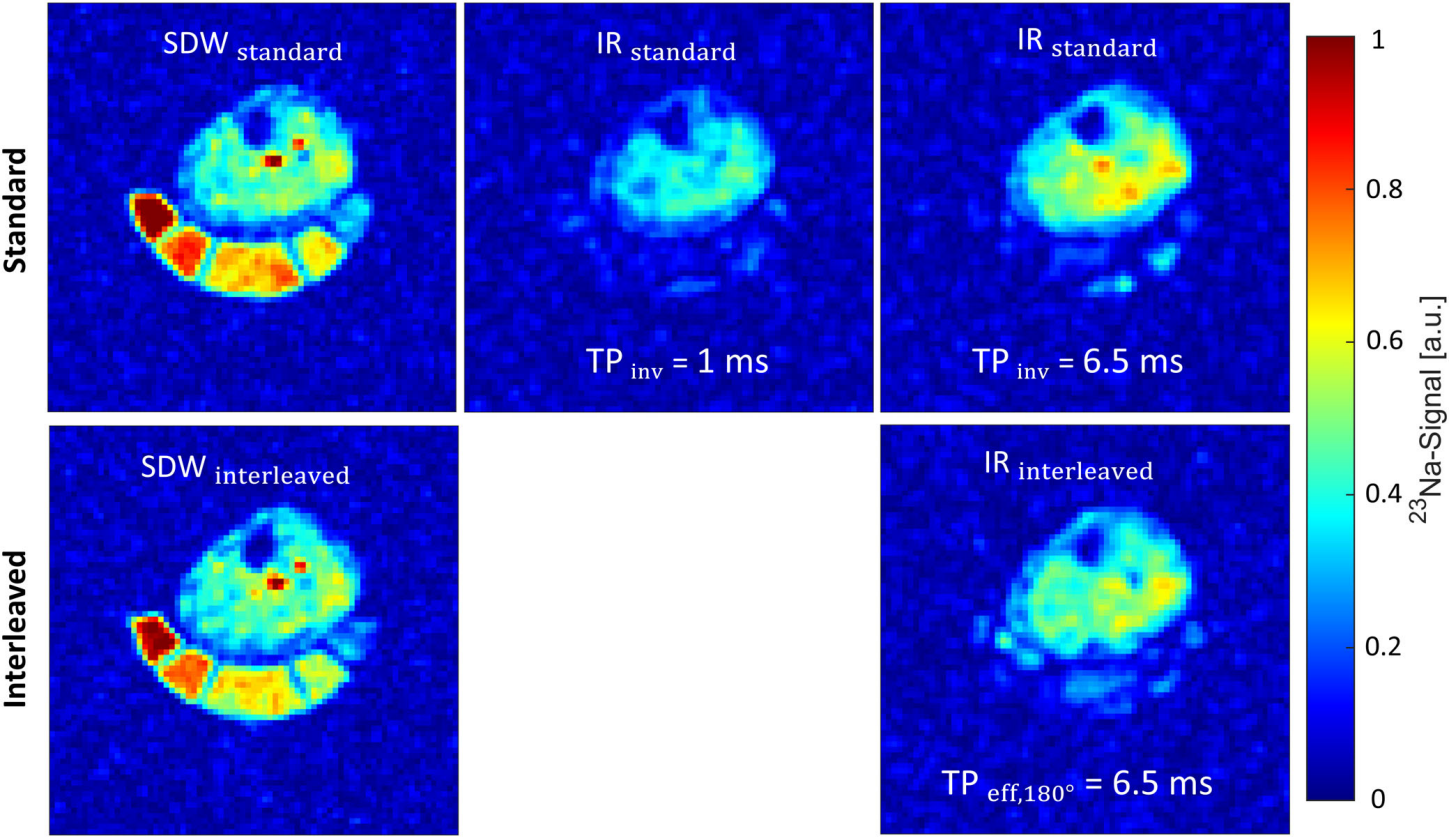
In vivo measurement results for one volunteer (Female 03) for all investigated sequences. As with the phantom measurement, there is a slight difference in signal intensity, especially in the reference holder, between the standard and the interleaved SDW image. Between the SDW images, no relevant changes in signal intensity or contrast are observable in the muscle tissue. For longer effective inversion pulse lengths, suppression artifacts in the reference phantoms caused by *B*_0_ inhomogeneities become slightly stronger. Furthermore, in muscle the signal increase with longer effective inversion pulse lengths is particularly noticeable. Compared to the standard IR sequence with TP_inv_ = 6.5 ms, the interleaved IR sequence shows almost identical results.

The quantitative analysis of the signal in the muscle tissue is shown in Figure 6. No significant differences were found between the SDW signal intensity of the standard and the interleaved sequence, neither for the whole muscle nor for specific ROIs. Concerning the investigated IR sequences, the standard IR with TP_inv_ = 1 ms provided significantly lower signal (*p* < 0.01). No significant differences were found between the intensity of the standard IR signal with TP_inv_ = 6.5 ms and the IR signal of the interleaved sequence. All mean values of signal intensity for the different sequences can be found in Table S1.

**FIGURE 6:**
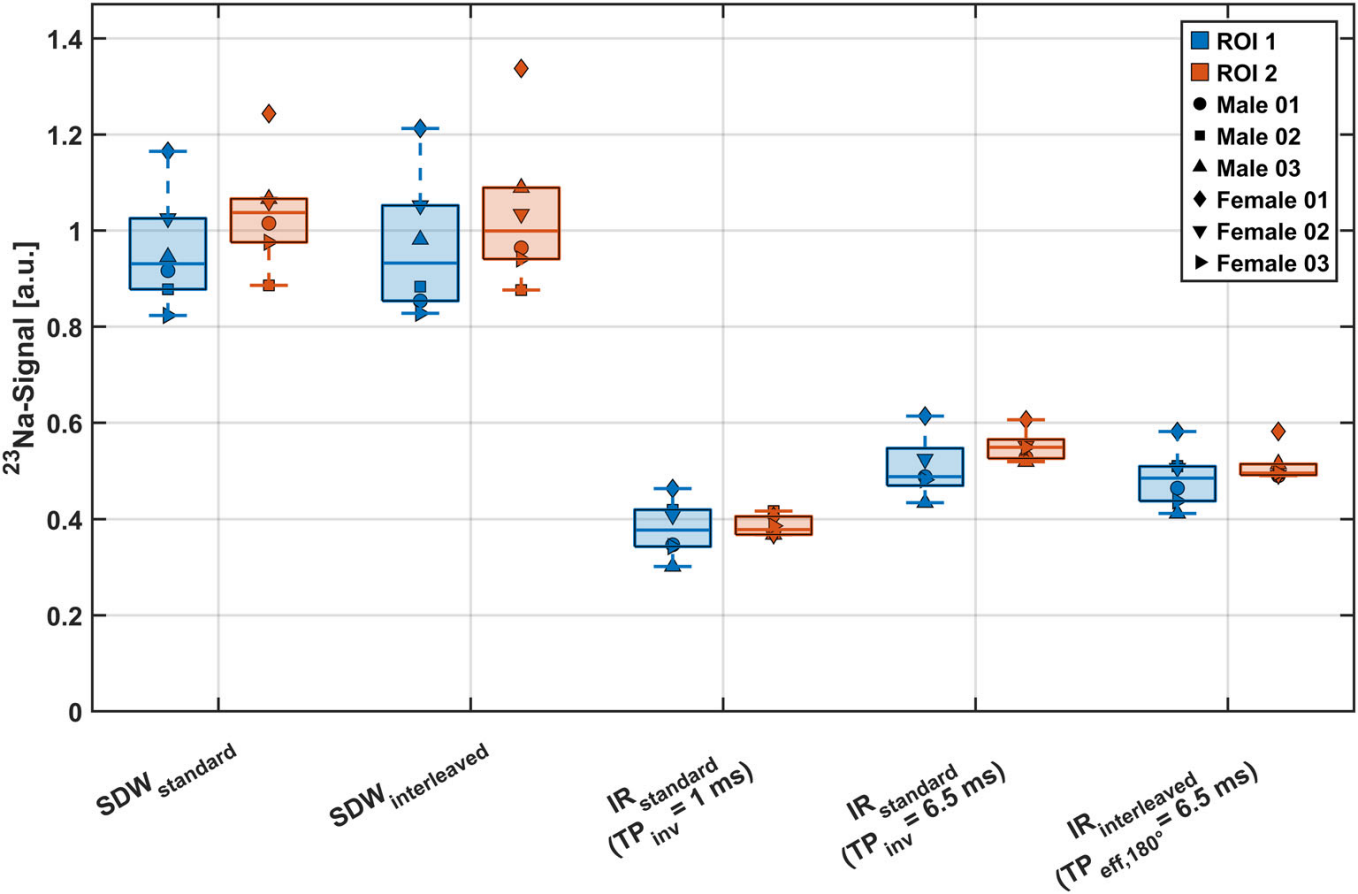
Boxplots of the signal intensity values of the different investigated sequences for the two examined ROIs. All signal intensities were normalized to the mean value of the whole muscle of the standard SDW image. ROI 1 corresponds to the gastrocnemius lateralis, the gastrocnemius medialis, and the soleus muscle. ROI 2 corresponds to the tibialis anterior, the peroneus longus, and the extensor digitorum longus muscle. No significant differences were found between the signal intensity of the standard sequences compared to the interleaved sequence for corresponding sequence parameters.

## 4 Discussion

This work introduces a novel interleaved SDW/IR ^23^Na MRI sequence that provides both SDW and IR image data within a single sequence without increasing acquisition time. Consequently, an additional IR acquisition can be incorporated to study protocols without additional scan time, or, if both contrasts are already included in the study protocol, the total acquisition time can be significantly reduced. For identical sequence parameters, neither for the SDW contrast nor the IR contrast significant differences in the muscle ^23^Na signal intensity were observed between the interleaved SDW/IR sequence and the corresponding standard sequences. For the fast *T*_1_‐relaxation of muscle tissue, no signal changes resulting from a different *T*_1_‐weighting were observable. In addition to the reduced scan time, the interleaved acquisition of SDW and IR contrasts in the developed SDW/IR sequence eliminates the need for co‐registration, since both contrasts are acquired at the same time. This is particularly beneficial for studies investigating triggered changes in the muscle metabolism, for example through exercise [28, 29]. Furthermore, the interleaved acquisition also avoids sodium distribution drifts between SDW and IR acquisition, which can result from physiological changes in muscle aTSC during prolonged supine position [30].

For the same effective inversion pulse duration, only minor differences were observed between the standard IR and the interleaved SDW/IR sequence. As shown in other publications [7, 13, 31], long inversion pulses improve the IR contrast for non‐suppressed compartments at the cost of higher sensitivity to *B*_0_ inhomogeneities. In this work, the same effects were also shown for corresponding effective inversion pulse lengths covering two short 90° pulses with an intermediate signal acquisition inserted in between. Depending on the *T*_1_ as well as *T*_2_* relaxation characteristics of the tissue, the effects are slightly more pronounced in the interleaved IR acquisition. Furthermore, as shown by a former study investigating long inversion pulses for ^23^Na calf muscle IR MRI [13], our measurements also showed a slight change in contrast between arteries and muscle compared to the standard IR with short inversion pulse duration. However, no change of contrast between different muscle groups was observed. For the same effective inversion pulse length, no significant differences were observable between the IR signal intensities of the interleaved SDW/IR and the standard IR sequence.

In the interleaved SDW/IR sequence, we aimed to preserve the image quality of the SDW data set and get an additional IR data set without additional scan time. For this purpose, the SDW readout time of *T*_RO_ = 5 ms, which lies at the lower bound of the typical range of 5–10 ms for readout times used in ^23^Na studies at our institute [10, 11, 32, 33], was kept constant for both sequences. In general, longer readout times could improve SNR; however, for the very fast relaxation behavior of ^23^Na nuclei, longer readout times lead to lower effective spatial resolution due to broader point spread functions. For the fast biexponential transverse relaxation of ^23^Na in muscle tissue [4], relative SNR for small objects is already close to optimal [20]. Furthermore, for the interleaved SDW/IR sequence, the choice of the SDW readout time results in a trade‐off between the SNR of the SDW image data and the sensitivity to *B*_0_ inhomogeneities of the fluid suppression in the IR contrast. Consequently, the SDW readout time has to be adapted according to the focus and needs of the specific measurement or study. In general, depending on the specific requirements, the interleaved SDW/IR sequence parameters can be optimized for one of the two contrasts.

Furthermore, it might also be possible to split the inversion pulse into two pulses with different pulse lengths and different flip angles. For example, the first pulse can be very short with a lower flip angle to also acquire very fast decaying signal components [24, 34, 35] with a corresponding longer second pulse with a higher flip angle to complete the 180° inversion. This, however, might influence the calculation of TI as well as the sequence behavior with regard to *B*_0_ and *B*_1_ inhomogeneities and should be explored in further studies.

In contrast to former works using a similar procedure for the interleaved acquisition of multiple contrasts of ^1^H MRI [36], in our work the series of 90° RF pulses did not result in any problems regarding stimulated echoes. Due to the very rapid transverse magnetization decay of ^23^Na, even the signal of saline which is the slowest relaxing compartment (*T*_2_ = 56 ms [13]) decays to ≤ 0.5% after less than two TRs for the chosen TR of 150 ms of the interleaved SDW/IR sequence. Furthermore, the sequence timing is chosen to suppress saline, which therefore does not result in transverse magnetization for this compartment after the excitation pulse. The transverse magnetization of muscle (*T*_2s_ < 3.6 ms [4], *T*_2l_ < 26.6 ms [4]) has already decayed after one TR to ≤ 0.5%. In addition to the fast signal decay, we use RF spoiling between every TR and spoiler gradients after the last readout of every TR. Consequently, no stimulated echo effects were recognized or are expected for our sequence.

In this work, we demonstrated the interleaved SDW/IR sequence using a density‐adapted 3D radial readout scheme [20]. However, as the approach does not affect the readout itself, the method can also easily be implemented for other readout schemes commonly used for ^23^Na MRI, such as twisted projection imaging (TPI) [37, 38], 3D cones [39, 40] or fermat‐looped orthogonally encoded trajectories (FLORET) [41]. Furthermore, many studies about human calf muscle investigate ^23^Na and ^1^H MRI data [9, 10, 11]. As the sequence timing compared to standard ^23^Na SDW or IR sequences is only slightly changed, the sequence idle times still can be used for interleaving ^23^Na MRI with ^1^H MRI to obtain data for tissue segmentation [15] or further anatomical examinations [16] and tissue parameters [17] to shorten the total MR protocol scan time. In the conducted measurements using a dual‐tuned ^23^Na/^39^K leg RF coil, the choice of pulse sequence parameters was not limited by SAR. However, SAR limitations might be an issue for other coils or in other body regions. In cases where common SDW acquisitions already are near the SAR limitations, sequence parameters relevant for the SDW contrast, particularly TR and TP_exc_, have to be changed to run the interleaved SDW/IR sequence. On the other hand, compared to the standard IR sequence, SAR remains unchanged for the interleaved SDW/IR sequence.

## 5 Conclusion

In conclusion, the proposed interleaved SDW/IR sequence provides the opportunity to shorten scan time of ^23^Na MRI protocols by including an IR contrast into SDW ^23^Na MRI without extending measurement time. This is an important step to include both contrasts in time‐efficient MRI protocols facilitating the application in clinical research studies.

## Funding

This work was supported by project Deutsche Forschungsgemeinschaft, 500888779/RU5534.

## Conflicts of Interest

The authors declare no conflicts of interest.

## Supporting information


**Figure S1:** Measurement results for all investigated sequences with TR = 360 ms. Compared to the standard SDW image (A), the interleaved SDW image (C) presents a slightly reduced signal for all compartments without any changes of the image contrast (E). The IR images (B, D) present a very good fluid suppression and the interleaved sequence generates signal intensities that are almost identical to those produced by the standard sequence.
**Figure S2:** To investigate the influence of the SDW readout and the corresponding rewinder gradients on the IR image of the interleaved SDW/IR sequence (A, B), phantom measurements were performed using an additional sequence with exact the same sequence timing as the interleaved sequence but without SDW readout and corresponding rewinder gradients (C). Compared to the difference between two consecutive scans with the interleaved sequence (D), only slightly increased differences in regions affected by *B*_0_ inhomogeneity‐related artifacts were found between the interleaved and the modified interleaved sequence (E).
**Figure S3:** mrm70536‐sup‐0001‐Supinfo.docx. ^23^Na IR measurement results of all six healthy volunteers. Signal originating from the reference phantoms with saline solution is well suppressed in all IR measurements with only minor subject‐dependent suppression artifacts that are slightly more pronounced at longer effective inversion pulse lengths in regions with higher off‐resonances. Due to scan time restrictions, for volunteer Male 02 no standard IR measurement with TP_inv_ = 6.5 ms is available.
**Table S1:** Mean signal intensities over all six volunteers for all investigated sequences. The signal intensities of the SDW images were corrected for the higher spatial resolution by the factor (Δ*x* ^3^)_IR_/(Δ*x* ^3^)_SDW_ and all values were normalized to the mean value of the whole muscle of the standard SDW image.

## Data Availability

The data that support the findings of this study are available on request from the corresponding author. The data are not publicly available due to privacy or ethical restrictions.
