# Supplementary material for: Time‐Efficient Interleaved Spin Density‐Weighted and Inversion Recovery 23Na MRI of the Human Calf Muscle at 7 T
Source: Magn Reson Med. 2026 Jul 21;96(6):2675–84. doi: 10.1002/mrm.70536 (PMC13619856; doi:10.1002/mrm.70536)
Supplement: Supplementary file 1 [file MRM-96-2675-s001.docx]

**Supplementary Material**

***Supporting Information Figure S1:*** *Measurement results for all investigated sequences with TR = 360 ms. Compared to the standard SDW image (A), the interleaved SDW image (C) presents a slightly reduced signal for all compartments without any changes of the image contrast (E). The IR images (B, D) present a very good fluid suppression and the interleaved sequence generates signal intensities that are almost identical to those produced by the standard sequence.*

***Supporting Information Figure S2:*** *To investigate the influence of the SDW readout and the corresponding rewinder gradients on the IR image of the interleaved SDW/IR sequence (A, B), phantom measurements were performed using an additional sequence with exact the same sequence timing as the interleaved sequence but without SDW readout and corresponding rewinder gradients (C).* *Compared to the difference between two consecutive scans with identical sequence settings of the interleaved sequence (D), only slightly increased differences in regions affected by B_0_ inhomogeneity-related artifacts were found between the interleaved and the modified interleaved sequence (E).*

***Supporting Information Figure S3:*** *^23^Na IR measurement results of all six healthy volunteers. Signal originating from the reference phantoms with saline solution is well suppressed in all IR measurements with only minor subject-dependent suppression artifacts that are slightly more pronounced at longer effective inversion pulse lengths in regions with higher off-resonances.* *Due to scan time restrictions, for volunteer Male 02 no standard IR measurement with TP_inv_ = 6.5 ms is available.*

***Supporting Information Table S1:*** *Mean signal intensities over all six volunteers for all investigated sequences.* *The signal intensities of the SDW images were corrected for the higher spatial resolution by the factor (Δx³)IR/(Δx³)SDW and all values were normalized to the mean value of the whole muscle of the standard SDW image.*

| Sequence | SDW | | IR | | |
| --- | --- | --- | --- | --- | --- |
|  | Standard | Interleaved | Standard  (TP_inv_ = 1 ms) | Standard  (TP_inv_ = 6.5 ms) | Interleaved  (TP_eff,180°_ = 6.5 ms) |
| ROI 1 | 0.96 ± 0.12 | 0.97 ± 0.15 | 0.38 ± 0.06 | 0.52 ± 0.06 | 0.48 ± 0.05 |
| ROI 2 | 1.04 ± 0.12 | 1.04 ± 0.16 | 0.38 ± 0.02 | 0.55 ± 0.03 | 0.51 ± 0.03 |
| ROI1+ROI2 | 1.00 ± 0.11 | 1.01 ± 0.15 | 0.38 ± 0.04 | 0.53 ± 0.05 | 0.50 ± 0.04 |
